# Supplementary material for: Geometric cues stabilise long-axis polarisation of PAR protein patterns in C. elegans
Source: Nat Commun. 2020 Jan 27;11:539. doi: 10.1038/s41467-020-14317-w (PMC6985163; doi:10.1038/s41467-020-14317-w)
Supplement: Supplementary file 1 — Description of Additional Supplementary Files [file 41467_2020_14317_MOESM1_ESM.pdf]

Supplementary Movies:

All Supplementary Movies were produced from image sequences from FEM (COMSOL) simulations. The image sequences were wrapped using the free program ffmpeg (https://www.ffmpeg.org/).

Supplementary Movie 1 (M2d_1.mp4) shows a simulation of our 2d model, specifically the time evolution of membrane concentrations ($A_1 + A_{12}$ in red and P in blue) for the cytosolic reactivation and diffusion $\lambda = 0.05\mathrm{s}^{-1}$ and $\mathrm{D_{cyt}} = 30\mathrm{\mu m^2 s^{-1}}$. All other parameters were set according to Table 1 in the main manuscript.
Membrane concentrations in the 2d ellipse are given in numbers of 5 particles per unit length (i.e. a line density).

Supplementary Movie 2 (M2d_2.mp4) shows a simulation of our 2d model, specifically the time evolution of membrane concentrations ($A_1 + A_{12}$ in red and P in blue) for the cytosolic reactivation and diffusion $\lambda = 0.3\mathrm{s}^{-1}$ and $\mathrm{D_{cyt}} = 30\mathrm{\mu m^2 s^{-1}}$. All other parameters were set according to Table 1 in the main manuscript.
Membrane concentrations in the 2d ellipse are given in numbers of 5 particles per unit length (i.e. a line density).

Supplementary Movie 3 (M2d_3.mp4) shows a simulation of our 2d model, specifically the time evolution of membrane concentrations ($A_1 + A_{12}$ in red and P in blue) for the cytosolic reactivation and diffusion $\lambda = 0.3\mathrm{s}^{-1}$ and $\mathrm{D_{cyt}} = 30\mathrm{\mu m^2 s^{-1}}$. All other parameters were set according to Table 1 in the main manuscript.
Membrane concentrations in the 2d ellipse are given in numbers of 5 particles per unit length (i.e. a line density).

Supplementary Movies 4 to 8 (M3d_1.mp4 to M3d_5.mp4) show simulations of the 3d model, specifically, the time evolution of the membrane concentration of $A_1 + A_{12}$ (rainbow colour code) starting with an aPAR dominant membrane state. Different movies show different geometries and different cytosolic parameters.
Parameters were set according to Table 4 in the main text for the prolate geometry and according to Supplementary Table 4 for the oblate geometry.

Supplementary Movie 4 shows, in prolate geometry, the establishment of steady state long-axis polarisation from onset on. The cytosolic reactivation and diffusion are set to $\lambda = 0.05\mathrm{s}^{-1}$and $\mathrm{D_{cyt}} = 50\mathrm{\mu m^2} s^{-1}$, all other parameters are given in Table 4 of the main text.

Supplementary Movie 5 shows, in prolate geometry, the establishment of short-axis polarisation at onset which then rotates to a steady state long-axis polarisation. The cytosolic reactivation and diffusion are set to $\lambda = 0.3\mathrm{s}^{-1}$and $\mathrm{D_{cyt}} = 10\mathrm{\mu m^2} s^{-1}$, all other parameters are given in Table 4 of the main text.

Supplementary Movie 6 shows, in oblate geometry, the establishment of short-axis polarisation at onset which then rotates to a steady state long-axis polarisation. The cytosolic reactivation and diffusion are set to $\lambda = 0.3\mathrm{s}^{-1}$and $\mathrm{D_{cyt}} = 18\mathrm{\mu m^2} s^{-1}$, all other parameters are given in Supplementary Table 4 of the main text.

Supplementary Movie 7 shows, in oblate geometry, the establishment of steady state long-axis polarisation from onset on. The cytosolic reactivation and diffusion are set to $\lambda =$

$0.03\mathrm{s}^{-1}$and $\mathrm{D_{cyt}} = 50\mathrm{\mu m}^2 s^{-1}$, all other parameters are given in Supplementary Table 4 of the main text.

Supplementary Movie 8 shows, in oblate geometry, the establishment of steady state short-axis polarisation from onset on. The cytosolic reactivation and diffusion are set to $\lambda = 0.2 s^{-1}$and $\mathrm{D_{cyt}} = 3\mathrm{\mu m}^2 s^{-1}$, all other parameters are given in Supplementary Table 4 of the main text.
